# Biocontrol Potential of Grapevine Endophytic and Rhizospheric Fungi Against Trunk Pathogens

**DOI:** 10.3389/fmicb.2020.614620

**Published:** 2021-01-07

**Authors:** Isidora Silva-Valderrama, Diana Toapanta, Maria de los Angeles Miccono, Mauricio Lolas, Gonzalo A. Díaz, Dario Cantu, Alvaro Castro

**Affiliations:** ^1^UC Davis Chile Life Sciences Innovation Center, Santiago, Chile; ^2^Department of Viticulture and Enology, University of California, Davis, Davis, CA, United States; ^3^Laboratorio de Patología Frutal, Facultad de Ciencias Agrarias, Universidad de Talca, Talca, Chile

**Keywords:** biological control, fungal antagonism, co-culture experiments, grapevine trunk diseases, fungal endophyte

## Abstract

Grapevine Trunk Diseases (GTDs) are a major challenge to the grape industry worldwide. GTDs are responsible for considerable loss of quality, production, and vineyard longevity. Seventy-five percent of Chilean vineyards are estimated to be affected by GTDs. GTDs are complex diseases caused by several fungi species, including members of the Botryosphaeriaceae family and *Phaeomoniella chlamydospora*, considered some of the most important causal agents for these diseases in Chile. In this study, we isolated 169 endophytic and 209 rhizospheric fungi from grapevines grown under organic and conventional farming in Chile. Multiple isolates of *Chaetomium* sp., *Cladosporium* sp., *Clonostachys rosea, Epicoccum nigrum, Purpureocillium lilacinum*, and *Trichoderma* sp. were evaluated for their potential of biocontrol activity against *Diplodia seriata*, *Neofusicoccum parvum*, and *Pa. chlamydospora.* Tests of antagonism were carried out using two dual-culture-plate methods with multiple media types, including agar containing grapevine wood extract to simulate *in planta* nutrient conditions. Significant pathogen growth inhibition was observed by all isolates tested. *Clonostachys rosea* showed 98.2% inhibition of all pathogens in the presence of grapevine wood extract. We observed 100% pathogen growth inhibition when autoclaved lignified grapevine shoots were pre-inoculated with either *C. rosea* strains or *Trichoderma* sp. Overall, these results show that *C. rosea* strains isolated from grapevines are promising biocontrol agents against GTDs.

## Introduction

Grapevine trunk diseases (GTDs) are a major challenge to viticulture worldwide because they compromise the productivity and longevity of grapevines (*Vitis vinifera* L.) and increase production costs (Munkvold et al., 1994; Bertsch et al., 2013; Kaplan et al., 2016; Gramaje et al., 2018). GTDs are one of the main phytosanitary problems of the grape industry also in Chile (Auger et al., 2004; Díaz et al., 2011b). Chile is the first and fourth largest grape and wine exporter globally, respectively (Felzensztein, 2014; Pizarro, 2018; USDA Foreign Agricultural Center, 2019). In 2013, about 22% of the commercial vineyards in Chile showed symptoms of GTDs (Díaz et al., 2013; Latorre, 2018).

GTDs are a group of diseases affecting the grapevine trunk and internal tissue (Mugnai, 2011), resulting in foliar symptoms, cankers, and dieback of the plant (Gramaje et al., 2018). These diseases are caused by a wide range of fungi (Trouillas et al., 2010; Gramaje and Armengol, 2011; Úrbez-Torres, 2011; Augusti-Brisach and Armengol, 2013; Lombard et al., 2014; Gramaje et al., 2018) that often infect established grapevines through wounds produced during winter pruning (Rolshausen et al., 2010). GTDs can also spread during plant propagation (Aroca et al., 2010; Gramaje and Armengol, 2011), with infections found in dormant wood cuttings and young grafted plants (Waite and Morton, 2007; Gramaje and Armengol, 2011; Billones-Baaijens et al., 2013). In Chile, as in other viticulture areas, the most common microorganisms isolated from arms and trunks of grapevines with symptoms of GTDs are ascomycetous fungi and include *Phaeomoniella* (Pa.) *chlamydospora, Diplodia seriata* De Not., and *Neofusicoccum parvum* (Auger et al., 2004; Díaz et al., 2011a; Besoain et al., 2013; Díaz and Latorre, 2013; Díaz and Latorre, 2014).

Currently, there are no curative treatments against GTDs besides surgical removal of the infected organs (Surico et al., 2006; Wagschal et al., 2008; Gramaje et al., 2018; Mondello et al., 2018; Sosnowski and Mundy, 2018). GTDs are managed mostly by practices that aim to prevent infections (Gramaje et al., 2018; Mondello et al., 2018). Widely adopted preventive practices include late pruning (Petzoldt, 1981; Munkvold et al., 1994), double-pruning (Weber et al., 2007), and the application of protectants on fresh pruning wounds (Sosnowski and Mundy, 2019) as benomyl and tebuconazole (Bester et al., 2007), inorganic compounds as boric acid (Rolshausen and Gubler, 2005), or natural antifungal compounds as organic extracts (Mondello et al., 2018). Manual applications of these formulations as paints are effective, but costly and time-consuming, while spray applications are difficult due to the small surface and orientation of pruning wounds (Rolshausen et al., 2010; Wightwick et al., 2010; Bertsch et al., 2013). In addition, no genetic resistance against GTDs has been found in the grapevine germplasm (Surico et al., 2006; Wagschal et al., 2008).

Biocontrol of GTDs using microorganisms is a promising alternative. For example, *Trichoderma* spp. are effective as a protectant of pruning wounds (John et al., 2004; Halleen et al., 2010; Mondello et al., 2018). The goal of our work was to identify microorganisms with biocontrol potential among the natural microbial inhabitants of grapevines. Endophytes are microorganisms that inhabit and colonize the internal plant tissue without causing visible damage or illness in the host (Petrini, 1991; Hirsch and Braun, 1992; Stone et al., 2000; Schulz and Boyle, 2005). These microorganisms are known to mediate plant-environment as well as plant-pathogen interactions (White et al., 1997; Zabalgogeazcoa, 2008). The contribution of different epiphytes and endophyte species to plant defenses has been widely documented (Azevedo et al., 2000; Arnold et al., 2003; Pieterse et al., 2014). Plant defense induction and antibiotic substance production that inhibits the growth of pathogens and pests (Mousa and Raizada, 2013), by endophytic fungi (Arnold et al., 2001; Kaul et al., 2012), bacteria (Hardoim et al., 2008), viruses (Lehtonen et al., 2006), and insects (Azevedo et al., 2000) have been reported. The rationale behind focusing on endophytes in the search of effective biocontrol agents against GTDs was two-fold (Wicaksono et al., 2017). First, grapevine endophytes survive naturally inside this plant; therefore, these isolates, once applied, should have better chances to successfully colonize the internal tissue of the grapevine than biocontrol agents selected from other biological systems (Hardoim et al., 2008, 2015; López-Fernández et al., 2016). Second, endophytes share the same niche with plant pathogens; thus, in addition to plant-defense induction and antibiosis, they could also compete for space and nutrients with GTD pathogens (Zabalgogeazcoa, 2008; Aroca, 2013; Bacon and White, 2016).

Here we report the isolation and identification of endophytic and rhizospheric fungi from grapevines grown in commercial and non-commercial vineyards in Chile. From this collection, we selected antagonist candidates and evaluated them for growth inhibition activity against the main GTD fungal species found in Chile, in co-culture, and in planta assays, providing also a general inside of the mechanisms used for this. All of the above-mentioned, with the aim of finding potential biocontrol agents to control GTDs.

## Materials and Methods

### Sample Origin and Plant Material

Samples of grapevine (*Vitis vinifera* L.) cv. Cabernet Sauvignon and Chardonnay were collected from four commercial vineyards located in the central valleys in Chile under either organic or, conventional farming systems in May 2017 (Table 1). Samples of cv. País were collected in September 2017 from a vineyard where diseases are not managed, located in the Codpa Valley, Chile (Table 1). All plants sampled presented no symptoms of GTDs.

**TABLE 1:** Sample locations.

Vineyard	Variety	Location	Disease control	Planting year
Site 1	Chardonnay	−35°26′26.8764″S, −071°50′01.8600″W	conventional	
	Cabernet Sauvignon	−35°26′26.8764″S, −071°50′01.8600″W	conventional	
Site 2	Chardonnay	−34°42′53.3736″S, −071°02′20.5008″W	organic	2011
	Cabernet Sauvignon	−34°42′53.3736″S, −071°02′20.5008″W	organic	2009
Site 3		−33°44′592476″S, −070°56′18.6972″W	conventional	
Site 4	Cabernet Sauvignon	−33°44′592476″S, −070°56′18.6972″W	organic	2000
Site 5	País	−18°28′42.6″S, −070°05′16.2″W	none	1850

### Isolation of Endophytic Fungi

The isolation of endophytic fungi was performed following the methodology described in Pancher et al. (2012). Briefly, shoots (50 cm long) and roots were cut into 10-cm-long fragments. Fragments were surface disinfected by rounds of 2 min serial immersions in 90% ethanol, then 2% sodium hypochlorite solution, and 70% ethanol, followed by double-rinsing in sterile distilled water under laminar airflow. Absence of microbial growth on surface-sterilized shoots was confirmed by plating the distilled water from the last wash step on potato dextrose agar (PDA; BD-Difco) in Petri dishes, that were then incubated for 2 weeks at 25°C. After disinfection, fragments were further cut into 2.5 mm pieces. Each section was placed on Petri dishes (90-mm diameter), placing the vascular bundle toward the growing media, containing: (i) PDA (39 g L^–1^; BD-Difco), (ii) malt extract agar (MEA, 33.6 g L^–1^; BD-Difco), and (iii) plain agar (AA, 20 g L^–1^; Difco), each one with antibiotics (streptomycin, 0.05 g L^–1^, and chloramphenicol, 0.05 g L^–1^). All Petri dishes were incubated at 25°C for 7 to 10 days under 12 h of light and 12 of darkness. Different colonies were tentatively identified based in morphology (Barnett and Hunter, 1955). Pure cultures were obtained from hyphal tip transfer to PDA media and maintained at 5°C.

### Isolation of Rhizospheric Fungi

For each plant, 1.5 g of soil in direct contact with roots was carefully collected. In a laminar flow bench, 13.5 ml of sterile distilled water was added, before vigorous agitation for 20 min in a horizontal position. After 5 min of decantation, serial dilutions of the supernatant were made. 10^–3^ and 10^–4^ dilutions were used to inoculate PDA, MEA, and AA. To all media streptomycin, 0.05 g L^–1^ and chloramphenicol, 0.05 g L^–1^ were added. Plates were incubated for 7 to 14 days at 25°C.

### Taxonomic Characterization of the Fungal Isolates

DNA extraction from cultivable isolated fungi (*n* = 387 isolates) was performed as described in Morales-Cruz et al. (2015), with the following modifications. Mycelium from 7 to 21 days old fungal cultures were frozen with 3 mm metal beads in tubes at −80°C. Tubes were shaken vigorously with a vortex for 5 min at maximum speed. Disrupted mycelium were resuspended in 200 μL of nuclease-free sterile-distilled water and then homogenized in a vortex for 15 s. Mycelium was incubated at 100°C for 10 min, followed by a centrifugation step at 14500 rpm for 2 min. An aliquot of 10 μL of the supernatant was used for the PCR runs. A 1:20 or 1:50 dilution was made in case of PCR inhibition occurred. ITS sequences were PCR amplified using ITS1 (TCCGTAGGTGAACCTGCGG) and ITS4 (TCCTCCGCTTATTGATATGC) primers (White et al., 1990). A 25 μL PCR reaction was carried out using 2.5 μL 1X Thermopol reaction buffer, 0.5 μL of 10 mM dNTPs, 0.5 μL of 10 μM ITS forward and reverse primers, 0.125 μL (1.25U/50 μL) Taq DNA polymerase (Promega, United States) and 10 μL of sample supernatant as a template. PCR reaction was performed with an initial denaturing step at 95°C for 2 min, and 35 cycles of 95°C for 30 s, 52°C for 30 s (White et al., 1990), and 72°C for 1 min, followed by a final extension phase at 72°C for 5 min. The PCR product was purified and sequenced at Macrogen Inc., South Korea. Amplicon sequencing analysis was carried out with Geneious (R11.1). Taxonomic identities were determined with BLASTN using the UNITE database 7.2 (Nilsson et al., 2019).

### Pathogenic Fungal Strains and Control Antagonists Origin

Isolates of *Phaeomoniella chlamydospora* (#11 A), *Diplodia seriata* (N°117 Molina), *Neofussicoccum parvum* (N°156 Lolol) and the endophytic antagonist *Trichoderma* sp. (Altair 607 QR6 PB 6.0) were obtained from the Phytopathology Lab of Universidad de Talca. These isolates were obtained in 2017 from *V. vinifera* L. trunks as part of another project. Also, MAMULL (*Trichoderma gamsii* Volqui strain, *Bionectria ochroleuca* Mitique strain, *Hypocrea virens* Ñire strain, BioInsumos Nativa, Chile), TIFI (Giteniberica de Abonos, Spain), Tebuconazole 430 SC (SOLCHEM, concentrated suspension, Chile) were used as positive controls.

### Test of Fungal Antagonism

Initial assessment of antagonistic properties was conducted against *D. seriata* as pathogen. Further evaluations on selected antagonists were carried out using *D. seriata, N. parvum*, and *P. chlamydospora*. Agar disks from a 7-day old actively growing colony were used. Co-culture assays were performed placing a 5 mm agar disk on one side of the Petri dish with PDA (39 g L^–1^; Difco) or PA (200 g L^–1^ grapevine propagation material, 20 g L^–1^ agar) and on the opposite side a 5 mm agar disk containing the antagonist strain. Plates were incubated at 25°C for 7–28 days in darkness (Badalyan et al., 2002) using a randomized complete block design. Registered bioproducts MAMULL and TIFI were used as antagonistic controls. Pathogen growth area was evaluated at 7, 14, 21, and 28 days post-co-culture (Schindelin et al., 2012). Inhibition percentage was calculated using the pathogen growth area when was cultured alone (C) or in interaction with the antagonist (T) according to the formula I = [(C-T)/C) ^∗^ 100] (Thampi and Suseela, 2017).

**FIGURE 1:**
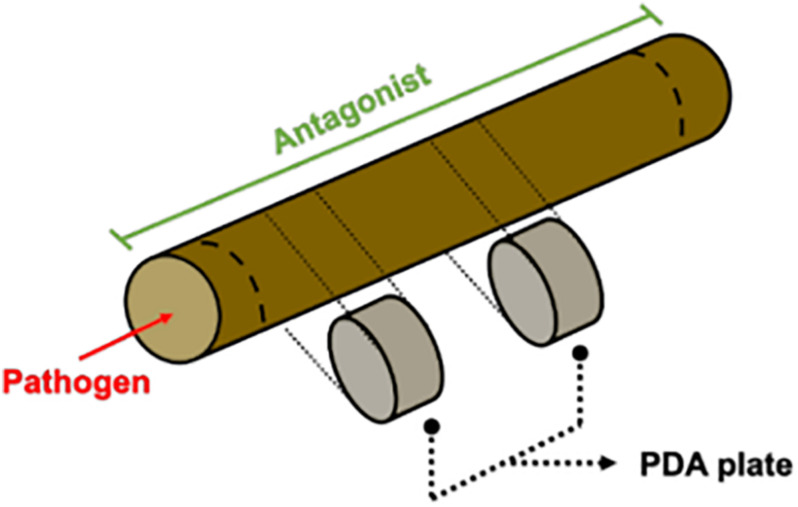
Annual shoots essay diagram. Shortly, shoots were inoculated with a spore suspension of the antagonist. Twenty-four hours later, the pathogen was inoculated on one side of the shoot and incubated in a humid chamber. After seven days, small pieces were taken and cultured in PDA plates.

An in planta assay was also performed. Annual shoots were used for the experimental set-up to verify the antagonistic potential shown in plate co-culture. Several preliminary evaluations were carried out in order to test variability caused by autoclave sterilization of pruning material, humid-chamber moist maintenance, type of inoculum and time needed for the pathogen to grow through the wood piece. Even though tissue was dead, the overall shoot matrix structure was conserved after autoclave sterilization (data not shown). Internode portions of dormant cuttings were cut in 4.5 cm length pieces and then used fresh or autoclaved for 25 min at 121 °C. Agar mycelium plugs were evaluated as inoculum. In 2 days, pruning material in contact with the pathogen and/or antagonist plugs were covered in the mycelium. As the inoculum type was too different from a field inoculum, a spore suspension solution was used to inoculate the wood pieces. Mycelium/spore mix suspension of the pathogens *D. seriata* and *N. parvum* were prepared by flooding 30 days old plant agar culture (PA; 200 g L^–1^ grapevine dormant cutting, 20 g L^–1^ agar) with sterile distilled water. In the case of the antagonists *Clonostachys rosea* (isolates CoS3/4.24, CoR2.15 and R31.6) a spore suspension adjusted to 1 × 10^7^ conidia mL^–1^ was used. Antagonist inoculation was carried out adding 40 μL of antagonist fresh spore suspension until it reached the woody stem cut end by capillarity. Tebuconazole (60 mL/100 L fields recommended doses; SOLCHEM, Chile) or sterile distilled water was applied in the same manner as controls. This experiment was carried out 5 times. Woody stem cuts were incubated in individual humid chambers for 24 h (Figure 1). Then, 10 μL of fresh pathogen mycelium/spore mix suspension was inoculated on the same side where the antagonist was inoculated previously and immediately placed in a horizontal position, preventing suspension diffusion. Incubation was carried out in humid chambers for 3–7 days. Afterward, the surface of the woody stem was disinfected by rubbing with 70% ethanol. With a hot sterile scalp, the bark and 0.5 cm of the woody stem ends were removed. Small pieces located at 1 and 2.5 cm from the inoculation point were collected and cultured in individual PDA plates at 25°C for 7 days. To evaluate the pathogen mycelium and spore suspension viability, 10 μL of the solution was inoculated in one side of the wooden piece as described above and immediately processed to obtain 3 mm pieces at 1 and 2.5 cm from the pathogen inoculation point. Every piece was cultured in PDA at 25°C for 7 days. The presence of the pathogen on PDA was evaluated under a light microscope.

### Test of Antagonist Mechanism

To characterize the mechanism of antagonism, the same experimental setup of co-culture was carried out on water agar (AA, 20 g L^–1^; Difco) with a microscope sterile slide covered by a thin layer of the same agar in its surface. Using a light microscope (MOTIC BA410), the sample was screened for loops of the antagonist hyphae around *N. parvum* and *D. seriata*, indicating mycoparasitism. This experiment was carried out 3 times. To determine antibiosis as the type of antagonist mechanism used, isolated fungi *E. nigrum* R39.1, *C. rosea* CoS3/4.4, and *Cladosporium* sp. B38d.2 were cultured in PDA plates (39 g L^–1^; Difco) over cellophane paper for 7 days. Cellophane paper with the fungal colony was then removed from the plate and a mycelium plug of *D. seriata* or *N. parvum* was placed in the center. Plates were incubated for 7 days at 25°C and pathogen growth was evaluated. This experiment was carried out three times.

### Statistical Analysis

Statistical analysis was conducted with GraphPad PRISM 8 (8.1.1 version, 2019).

## Results

### Isolation and Identification of Endophytic and Rhizospheric Fungi

**FIGURE 2:**
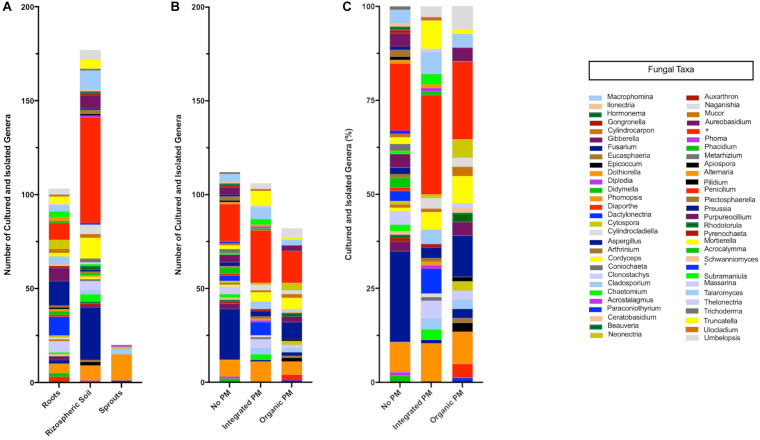
Taxonomic composition of the isolated fungi. Values are separated according to the source **(A)** and phytosanitary regime (pest management program, PM) **(B,C)**. Cultured-isolates identified only to family level Nectriaceae (+) and class level Dothideomycetes (^∗^) are also shown.

A total of 102 vineyard samples were collected to isolate endophytic and rhizospheric fungi associated with grapevines in Chile. Endophytic fungi were isolated from woody shoots, sprouts, and roots, while the rhizosphere ones were obtained from the soil in direct contact with the roots. Of these 102 samples, ninety were obtained from commercial vineyards in the central valleys of Chile and twelve from a vineyard in the Codpa Valley that has not been managed for disease protection for over 150 years. From these samples, a total of 221 and 166 morphologically different fungi were isolated from the commercial vineyards and the non-commercial Codpa Valley plants, respectively. Fungi were characterized taxonomically using ITS1 and ITS4 sequences. All fungal sequences were at least 98% identical to the best BLASTn hit in the UNITE database. We could assign taxonomy to a total of 300 isolates. The ITS sequence was discriminant at the species level for 227 isolates. The remaining were assigned to the corresponding genus or family. A total of 58 genera were represented, 37 and 38 among rhizospheric and endophytic fungi, respectively. As expected, below ground samples (rhizosphere and roots) were more diverse (56 genera) than sprouts and woody stems (5 genera) (Figure 2).

### Effect of Fungal Antagonists on the Growth of GTD Fungi in Co-culture

To identify potential biocontrol agents for further characterization, we screened all isolates for antagonistic activity against *D. seriata* (Supplementary Table 1), a ubiquitous GTD pathogen. Based on the results of this initial screen, a total of eight isolates were selected for further characterization: *Trichoderma* sp. Altair, *Epicoccum nigrum* R29.1, three isolates of *Clonostachys rosea* (R 31.6, CoR2.15 and CoS3/4.24), *Cladosporium* sp. B38d.2, *Chaetomium* sp. S34.6 and *Purpureocillium lilacinum* S36.1 (Supplementary Figure 1). Previous reports described the antagonistic ability of the isolated genera against other phytopathogens (Cota et al., 2009; de Lima Fávaro et al., 2012; Solano Castillo et al., 2014; Hung et al., 2015; Costadone and Gubler, 2016; Del Frari et al., 2019).

**FIGURE 3:**
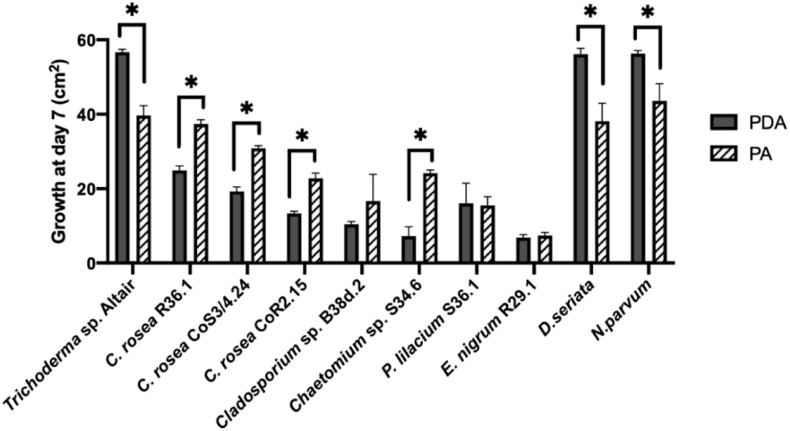
Comparison of the growth area of antagonists and pathogens in two media. Growth was measured after 7 days in PDA (potato dextrose agar) and PA (plant agar). Bars with asterisk are significantly different from the control (Paired *T* test, *P* < 0.001). Error bars represent the standard error of the mean, *n* = 5.

**FIGURE 4:**
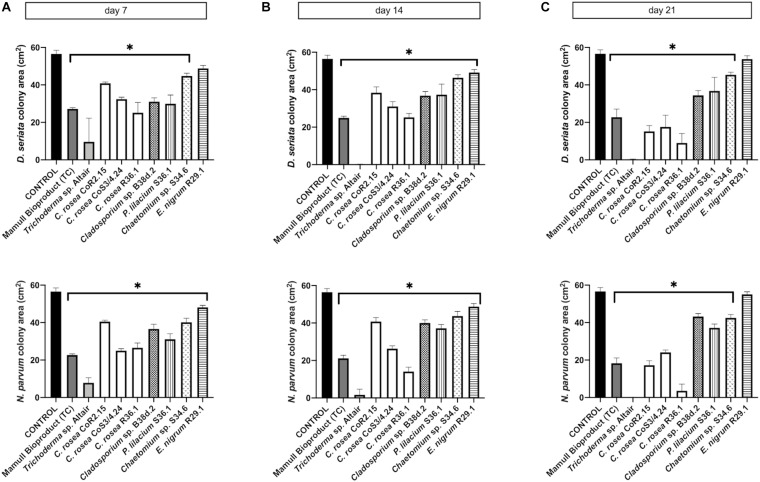
Colony area measured after **(A)** 7, **(B)** 14, and **(C)** 21 days of inoculation of *D. seriata* (upper graphics) and *N. parvum* (bottom graphics), when growing alone (control) or in co-culture with the antagonists in PDA. Bars with asterisk are significantly different to the control (Tukey’s test, *P* < 0.001). Error bars represent the standard error of the mean, *n* = 5.

**FIGURE 5:**
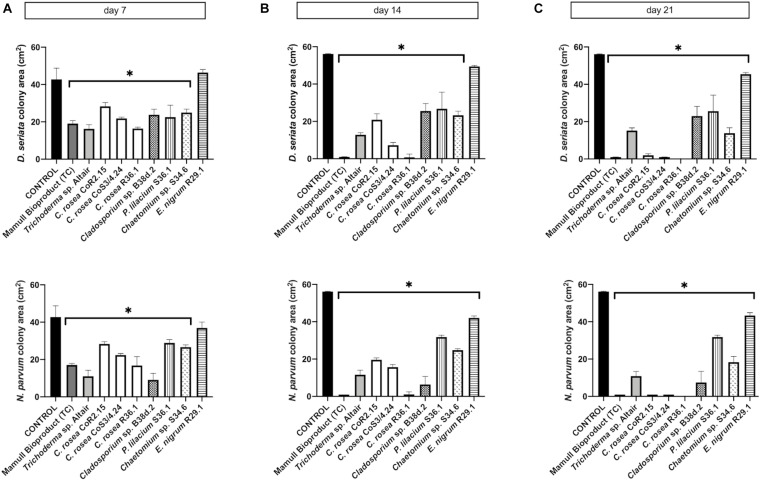
Colony area measured after **(A)** 7, **(B)** 14, and **(C)** 21 days of inoculation of *D. seriata* (top row) and *N. parvum* (bottom row), when growing alone (control) or in co-culture with the antagonists in PA. Bars with asterisk are significantly different to the control (Tukey’s test, *P* < 0.001). Error bars represent the standard error of the mean, *n* = 5.

To assess the antagonistic ability of the ten selected isolates, we co-cultured each one of them with *D. seriata* and *N. parvum*, two of the main fungi causing GTDs in Chile. Co-cultures were carried out on two different types of growth media: the commonly used potato dextrose agar (PDA) and a substrate made of agar and ground woody grapevine tissue aka, grapevine plant agar (PA)] that simulates in planta nutrient composition (Massonnet et al., 2017). Isolates displayed a wide range of growth rates, which often differed between PDA and PA (Figure 3). Interestingly, most endophytes, including all *C. rosea* isolates, grew faster on PA than PDA. Different growth rates reflected the patterns of inhibition of *D. seriata* and *N. parvum* (Figures 4, 5). The *Trichoderma* Altair isolate grew faster than the rest on PDA and reached its maximum inhibitory effect on both pathogens as early as day 7 in PDA. Growth inhibition only occurred upon physical contact between colonies of *Trichoderma* sp. and the pathogens. The faster growth on PA of the endophytes *Clonostachys, Chaetomium, Epicoccum*, and *Cladosporium* was associated with greater pathogen inhibition rates on this substrate compared to PDA, especially for the *Clonostachys* isolates. In PA, *C. rosea* overgrew the pathogen colony at least 7 days earlier than in PDA. All *C. rosea* strains inhibited over 98% pathogen growth in PA at day 21 (Figure 5). *Chaetomium* sp. S34.6 isolate inhibited pathogen growth by slowly growing in the plate until colony contact. By day 21 *Chaetomium* sp. S34.6 inhibited *D. seriata* and *N. parvum* growth by 59.1% and 86.75%, respectively, about two-fold the pathogen growth inhibition showed in PDA. Both species completely overgrew both pathogen colonies around 28 days. The antagonistic effect of *C. rosea* R36.1 and CoS3/4.24 occurred upon direct contact between colonies, which overgrew the pathogen colony within 21 days of growth. Instead, pathogen growth inhibition of *C. rosea* CoR2.15, *Purpureocillium lilacinum* S36.1, and *E. nigrum* R29.1 happened without evident physical contact between colonies. In PDA, *E. nigrum* produced a wide 0.8 to 1.2 cm orange-colored halo that was partially colonized only by *N. parvum* after 21 days of growth. The slow and limited growth of *Neofusicoccum parvum* was also visible in the halo produced by *Purpureocillium. Cladosporium* sp. B38d.2 showed an interesting difference in antagonist activity against *N. parvum* in PA, reaching its higher inhibition rate (Figure 5). When cultured with this pathogen, *Cladosporium* strongly sporulated, covering the entire plate, and stopped *N. parvum* early growth.

**FIGURE 6:**
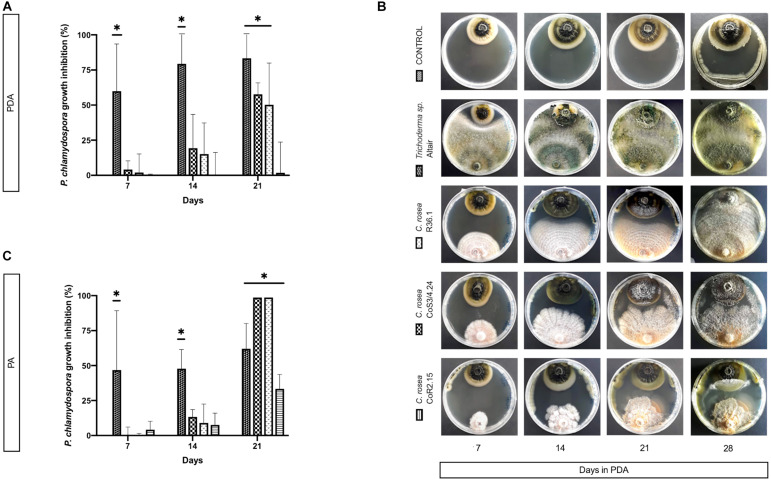
Colony area measured at day 7, 14, and 21 post-inoculation of the pathogen *P. chlamydospora* when cultured alone or with the antagonists: *C. rosea* CoR2.15, CoS3/4.24, R36.1 or *Trichoderma* sp. Altair. Growth area was evaluated in potato dextrose agar, PDA, **(A)** and **(B)** and in grapevine plant agar, PA, **(C)**. Bars with asterisk are significantly different to the control (Tukey’s test, *P* < 0.001). Error bars represent the standard error of the mean, *n* = 5.

On PA, *C. rosea* inhibited *P. chlamydospora* almost completely (99.9%). Interestingly, *C. rosea* growth first paused without evident contact between colonies (Figure 6) at day 7, but later, by 14 days, it overgrew completely the pathogen colony. Overgrowth was also observed with *Trichoderma* sp. Altair in PDA.

### Characterization of the Mechanisms of Antagonism

**FIGURE 7:**
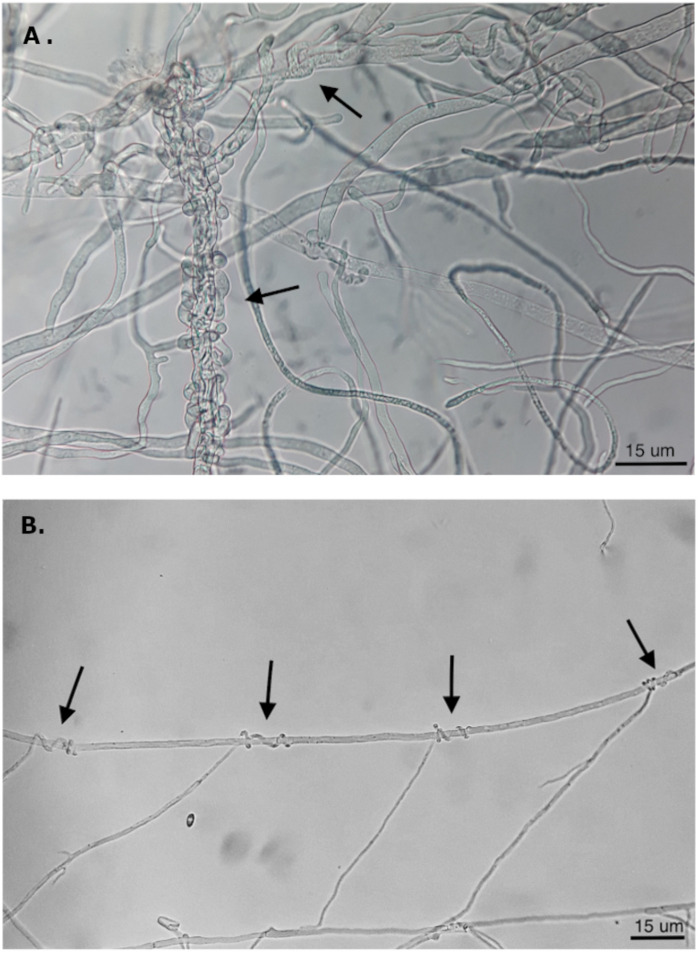
Hyphal coiling of **(A)** *Trichoderma* Altair against *D. seriata* and **(B)** *C. rosea* CoS3/4.24 around hyphae of *N. parvum* (magnification 400X).

The antagonistic activity of endophytic biocontrol agents can depend on the competition for nutrients and induced resistance in the plant, and/or direct interaction with the release of pathogen inhibitory compounds or mycoparasitism (White et al., 1997; Arnold et al., 2001; Köhl et al., 2015). During co-culture, isolates of *C. rosea* showed pathogen inhibition both before and after direct contact between colonies, suggesting that both mechanisms could underlie its antagonistic properties. To evaluate the mode of action of *C. rosea* and *Trichoderma* sp. Altair, we studied under a light microscope the mycelium in the zone of interspecific interaction. For *C. rosea* CoS3/4.24 and R36.1, hyphal coiling, a sign of mycoparasitism, was consistently observed in all co-cultures with *N. parvum* and *D. seriata* (Figure 7). Hyphal coiling was only occasionally found in *Trichoderma* sp. Altair.

**FIGURE 8:**
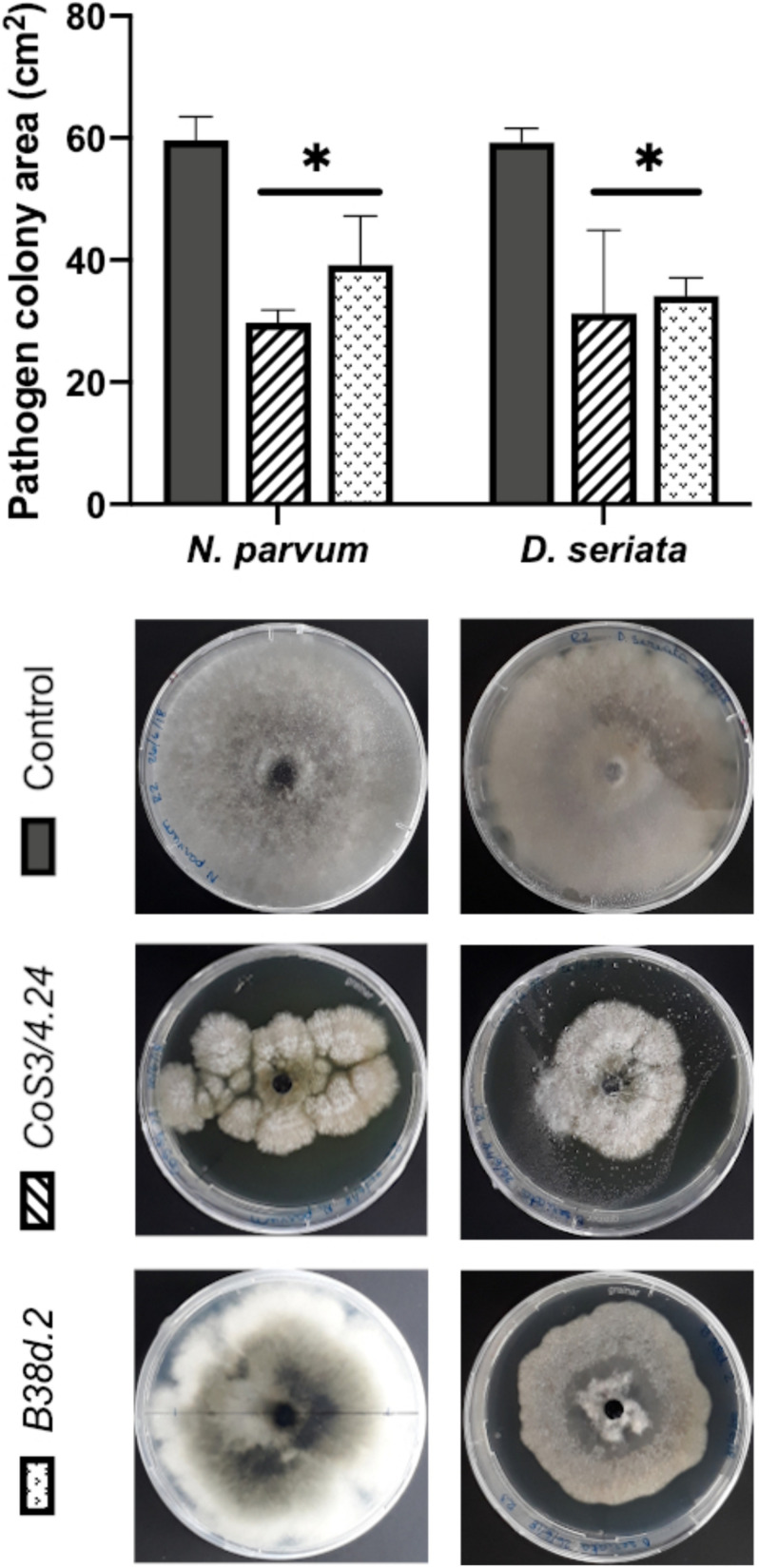
Pathogen growth over secondary metabolites produced by antagonists *C. rosea* CoS3/4.24, *Cladosporium* sp. B38d.2 in PDA. Bars with asterisk are significantly different to the control (Tukey’s test, *P* < 0.001). Error bars represent the standard error of the mean, *n* = 5.

When *C. rosea* rhizosphere strain CoS3/4.24 was co-cultured with *D. seriata* or *N. parvum*, pathogen growth terminated before direct contact with *C. rosea* in correspondence of the halo surrounding the antagonist. In this case, the inhibitory activity of *C. rosea* may depend on a secreted antibiotic compound. This was also observed when *Cladosporium* sp. B38d.2 was used as antagonist. To test the inhibitory activity of the C. rosea secretome, we inoculated *C. rosea* on a sterilized cellophane membrane overlaid on PDA and incubated for seven days. The cellophane membrane was shown to be permeable to metabolites secreted by fungi (Dennis and Webster, 1971; Chambers, 1993; Sharmini et al., 2004; Rodriguez et al., 2011). After removing the cellophane membrane together with the *C. rosea* mycelium, we inoculated the plates with pathogens and measured their growth in comparison with normal PDA. Pathogen growth was significantly reduced on plates previously incubated with *C. rosea*, likely due to the secreted metabolites that permeated through the cellophane membrane (Figure 8). The inhibition caused by the secreted metabolites of *C. rosea* CoS3/4.24 led to a 47.2% and 50.1% reduction in growth of *D. seriata* and *N. parvum*, respectively. In the case of Cladosporium sp., 34.26% and 42.46% inhibition was observed against *N. parvum* and *D. seriata*, respectively. Changes in the pathogen colony morphology were also observed, especially when in contact with *C. rosea* CoS3/4.24 isolate secondary metabolites. *N. parvum* colony turned into several flat independent colonies with undulate margins, while *D. seriata* grew as one colony with irregular shape.

### Effect of Fungal Antagonists on the Growth of GTD Fungi in One-Year old Grapevine Woody Shoots

**FIGURE 9:**
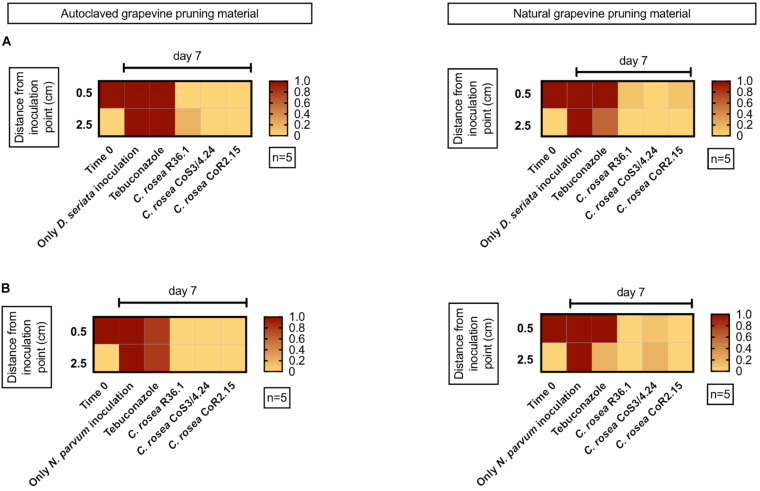
Presence of the pathogen *D. seriata* **(A)** and *N. parvum* **(B)** in autoclaved (left graphics) and natural (right graphics) grapevine pruning material pre-inoculated with the antagonist. In red is shown 100% recuperation of the pathogen.

As both growth and inhibition rates of GTD pathogens were significantly different in media containing grapevine annual shoot extract (plant agar, PA), we extended the testing of antagonism by using one-year-old lignified shoots (aka canes) as a substrate for co-cultures. We tested both sterile (autoclaved) and non-sterile canes. After 7 days, *C. rosea*, *N. parvum*, and *D. seriata* colonized completely the internal tissue of 4.5 cm-long autoclaved canes. The antagonists *C. rosea* strains were recovered in all pathogen co-inoculated samples after 7 days (Figure 9). No pathogen growth was observed at 0.5 cm from the pathogen inoculation point when treated with the antagonists. Interestingly, under the same conditions, Tebuconazole, a commercial synthetic fungicide, did not reduce *D. seriata* nor *N. parvum* growth.

We also performed the co-culture experiments on canes that were not subjected to autoclaving. Pathogens colonized the entire cane in 7 days in absence of any antagonist. In less than 0.1% and 10% of the co-culture assays, *N. parvum* and *D. seriata* were recovered from plant tissue previously inoculated with *C. rosea* isolates, respectively. In the case of CoS3/4.24 isolate, *N. parvum* and *D. seriata* growth inhibition was observed in 80% and 100% of the assays, respectively. In summary, the antagonistic potential of the *C. rosea* isolates shown in agar plate was confirmed in grapevine propagation material.

## Discussion

We isolated fungi from asymptomatic grapevines to find potential biocontrol agents against GTDs. As they share the same host with pathogens, these fungi may provide longer-lasting protection of grapevine tissues than biocontrol agents identified on other plant species (Zabalgogeazcoa, 2008; Latz et al., 2018). Three hundred eighty-seven different fungi and yeast were isolated and identified from multiple grapevine tissues and pest management systems. The observed diversity was limited to culturable fungi, since no cultivation-independent identification tools were applied. Taxa were determined solely based on the ITS sequence. Further validation using other informative sites, such as nu-SU-0817-59 and nu-SU-1196-39 (Borneman and Hartin, 2000) or TEF-1a (Ichi-Ishi and Inoue, 2005), would provide additional resolution for some of the isolates we were not able to characterize at the species level. As expected, rhizospheric soil showed to hold more fungal diversity than roots, and sprouts showed less cultivable diversity than any other sample. This was in agreement with previous studies using amplicon sequencing (Tan et al., 2017).

As the focus of this work was to find microorganisms able to colonize the grapevine persistently, we conducted this search during late Winter, at the beginning of the cold and wet season, when potentially beneficial microorganisms may compete with pathogens for the colonization of the host through pruning wounds (Arnold et al., 2003; Rolshausen et al., 2010; Travadon et al., 2016). Even if we could collect more samples from commercial vineyards than from the 150 year-old vines in the Codpa valley, the number of fungal taxa isolated from Codpa was higher than in commercial vineyards. The greater diversity found in Codpa might be due to the older age of the vines as well as the lack of pathogen control practices throughout the life of the vineyard, even if other cultural management practices as fertilization with animal manure have been done over generations.

All fungi we isolated, characterized, and tested, with the exception of ***Epicoccum nigrum*** showed a significant growth inhibition of ***N. parvum*** and ***D. seriata*** in co-cultures on both PDA and PA. The ***Trichoderma*** Altair isolate and all ***C. rosea*** strains completely overgrew both pathogens by day 21. This was also observed against the pathogen ***P. chlamydospora*** in PA. However, variable biocontrol efficacy was observed between different isolates of the same species, as reported in Inch and Gilbert (2007). For example, the rhizosphere isolate ***C. rosea*** CoS3/4.24 grew faster on media and overgrew the pathogen earlier than the other ***C. rosea*** isolates. In contrast, the endophytic isolates of ***C. rosea*** showed better inhibition of ***N. parvum*** in grapevine woodie shoots. The endophytic isolate of ***Cladosporium*** also displayed antagonism in co-culture, in particular against ***N. parvum*** on PA. Its inhibitory activity seemed to be due to the high sporulation rate and not to the rapid growth of the mycelium observed in others (Schöneberg et al., 2015). ***Cladosporium*** sp. produces a great amount of black, hydrophobic spores, and a small mycelium underneath the dense spore mass. On PA as well as PDA, ***Chaetomium*** sp. showed a significant reduction of growth of ***N. parvum*** and ***D. seriata***, although weaker than that of ***Trichoderma***. The antagonistic activity of ***Chaetomium*** may be due to a slow mycoparasitism. Hyphae of ***Chaetomium*** has been described to penetrate and coil around pathogen hyphae at day 30 of co-culture (Hung et al., 2015). Strains of ***Chaetomium*** have also shown antagonist activity against different pathogens as ***Phytophthora nicotianae*** (Hung et al., 2015), ***Rhizoctonia solani*** (Gao et al., 2005) and ***Fusarium oxysporum*** (Huu Phong et al., 2016) among others. Some strains presented antibiosis as an antagonist strategy, but mycoparasitism has been also described for this genus (Hung et al., 2015).

*Clonostachys rosea* showed limited antagonism at early stages of co-culture on artificial media and completely inhibited pathogen growth only after 21 days. Importantly, *C. rosea* was particularly effective against pathogen colonization of autoclaved woodie shoots. Fungal growth dynamics and therefore, the interaction between colonies are likely influenced by the type of media (Schöneberg et al., 2015), in particular when nutrient-rich media are compared with substrates poor in nutrients, such as PA and woodie tissue. It is worth noting that different isolates displayed different antagonistic activities depending on the substrate. For example, *C. rosea* isolates R36.1 and CoR2.15 showed higher pathogen inhibition than CoS3/4.24 on woodie shoots that were not autoclaved. Interestingly, R36.1 and CoR2.15 were endophytic, while CoS3/4.24 was isolated from the rhizosphere. Although we did not find the same pattern when autoclaved tissue was used, the different behavior of endophytic and rhizospheric isolates supports the overall strategy to search for potential biocontrol agents among the natural inhabitants of grapevines.

Generally recognized control mechanisms for fungal biocontrol agents are (1) competition for nutrients and space, (2) induced resistance in the plant, both consisting in an indirect interaction with the pathogen, (3) inhibition through antibiosis, and (4) mycoparasitism (Latz et al., 2018; Köhl et al., 2019). The formation of short loops of the antagonist’s hyphae around hyphae from another fungal species also called hyphal coiling (Barnett and Lilly, 1962; Assante et al., 2004; Gao et al., 2005). The coiling establishes an intimate contact with the parasitized hypha, penetrating the hypha and delivering antibiotic compounds and cell-wall degrading enzymes (Barnett and Lilly, 1962). This type of mycoparasitism has been commonly found in the genus *Trichoderma* (Howell, 2003; Benítez et al., 2004) and reported in *C. rosea* (Barnett and Lilly, 1962; Morandi et al., 2001). The Trichoderma sp. Altair isolate produced hyphal coils and also the *C. rosea* strains we tested. In all cases, we found a strong correlation between coiling and antagonism suggesting that mycoparasitism plays an important role in the interaction with the pathogens. In the case of *C. rosea* CoS3/4.24, a yellowish halo around the antagonist colony was present. Antibiosis was previously described for this species (Iqbal et al., 2017), but not all strains of the species show antibiotic production (Moraga-Suazo et al., 2016). Further studies should be performed with the *C. rosea* isolates to dissect the role of secondary metabolite production in pathogen growth inhibition and endophytic establishment in the grapevine as this might have important applications in agro-industrial areas (Karlsson et al., 2015). Direct interaction with the pathogen mode of action, as mycoparasitism and antibiosis, are highly desirable mechanisms for further production of commercial biocontrol agents, as they expose lower risks of human, plant and environmental toxicity (Köhl et al., 2019).

## Data Availability Statement

The datasets presented in this study can be found in online repositories. The names of the repository/repositories and accession number(s) can be found below: https://www.ncbi.nlm. nih.gov/genbank/, MN686237; https://www.ncbi.nlm.nih.gov/genbank/, MW077134; https://www.ncbi.nlm.nih.gov/genbank/, MW076517; https://www.ncbi.nlm.nih.gov/genbank/, MW130886; and https://www.ncbi.nlm.nih.gov/genbank/, MW130890.

## Author Contributions

IS-V was in charge of performing all experiments, developing and testing innovative evaluations, and writing the manuscript draft. DT gave strong support doing the experiments. MM gave guidance in the early steps of paper writing. ML and GD provided the pathogen fungi and *Trichoderma* sp. Altair isolates for evaluations, and some advice in project development and paper writing. DC gave invaluable and robust advice in paper writing. He also edited the manuscript a significant number of times. AC was in charge of the R&D department and the laboratory. He provided the first idea and gave guidance in every part of the project, generating the contacts for the development of this project. All authors contributed to the article and approved the submitted version.

## Conflict of Interest

The authors declare that the research was conducted in the absence of any commercial or financial relationships that could be construed as a potential conflict of interest.
